# Microcirculation and neutrophil-related cytokine concentrations are not altered around narrow diameter implants in T2DM patients during wound healing

**DOI:** 10.1007/s00784-022-04731-3

**Published:** 2022-10-13

**Authors:** Daniel Diehl, Doğan Kaner, Amelie Bockholt, Hakan Bilhan, Anton Friedmann

**Affiliations:** 1 Department of Periodontology, School of Dentistry, Faculty of Health, Witten/Herdecke University, Alfred-Herrhausen Str. 45, 58455 Witten, Germany; 2 Faculty of Health, Center for Biomedical Education and Research (ZBAF), Institute of Pharmacology and Toxicology, Witten/Herdecke University, Stockumer Straße 10, 58453 Witten, Germany

**Keywords:** Type 2 diabetes, Gingival perfusion, Gingival vascularization, Wound healing, Cytokines, PMN

## Abstract

**Objectives:**

The aim of this study was to assess the microcirculation and the expression patterns of wound-healing-related cytokines around narrow-diameter implants in type 2 diabetes mellitus (T2DM) and normo-glycemic patients.

**Materials and methods:**

A total of 31 patients, 16 of which diagnosed with T2DM (HbA1c > 6.5) and 15 normo-glycemic patients, received narrow diameter implants in the posterior mandible or maxilla. During the 3-month healing period, soft-tissue perfusion was monitored via laser Doppler flowmetry. Peri-implant fluid (PICF) was harvested and analyzed for concentrations of interleukin-1ß (IL-1ß), interleukin-23 (IL-23), interleukin-17 (IL-17), and granulocyte colony-stimulating factor (G-CSF) by a multiplex, bead-based immunoassay.

**Results:**

Microcirculatory perfusion patterns during wound healing exhibited no significant differences throughout the observation period. IL-1ß concentrations were expectedly elevated during the early phases of wound healing. At the first visit after surgery, IL-23 concentrations were significantly higher in implants of diabetic patients. This difference was diminished over the course of the observation period. For the other tested analytes, no differences were observable between both groups.

**Conclusion:**

Wound healing after implant surgery was similar in T2DM and healthy patients. Hydrophilic-surface titanium-zirconium implants with reduced diameter may be considered for implant therapy of diabetes mellitus type II patients.

**Registration number:**

NCT04630691 (clinicaltrials.gov).

## Introduction

Type 2 diabetes mellitus (T2DM) is a metabolic disorder characterized by high serum glycemic levels either due to insufficient insulin levels, defective function, or both [1]. In the last decade, the number of diabetic patients has nearly doubled and is now estimated to be at 285 million worldwide, 90% of which suffering from T2DM [2].

T2DM patients exhibit impaired wound healing and microcirculation, causing an increased risk of wound infection and exacerbating the inadequate host response to bacterial pathogens seen in periodontitis and peri-implantitis [3–7]. Known underlying mechanisms include decreased growth factor and cytokine production [8], impaired macrophage and neutrophil (PMN) function [9, 10], and accumulation of matrix-metalloproteases [11].

PMN constitute the first line of innate defense from the cellular immune system. Immediately after surgery-related injury inflicted on the tissue, an inflammatory reaction is initiated, and a variety of pro-inflammatory cytokines and chemokines mediate PMN chemotaxis to the affected area. One of the most abundant cytokines is interleukin-1ß (IL-1ß), which has emerged as a key regulator of pro-inflammatory tissue reaction and associated disorders [12].

Interleukin-17 is a cytokine family derived from T helper 17 (Th17) cells, which is known for its pro-inflammatory effects on both adaptive and innate host response, especially on PMN [13]. Moreover, the activated PMN themselves were shown to upregulate IL-17 expression in order to enhance the phagocytotic activity and the inflammatory phenotype differentiation [14]. Vice versa, the IL-17-mediated PMN activation upregulates the secretion of other chemotactic agents like CXC chemokines and the granulocyte colony stimulating factor (G-CSF) [15].

The activating cytokine repertoire needs to be maintained during the first phases of wound healing, for the PMN to function properly. This is achieved by PMN and macrophage-derived IL-23 [16]. Thus, IL-23 is known to be the main driver for Th17 polarization by CD4 + T-cells, which on the opposite constitutes the most sustainable source for IL-17 [17].

Under hyperglycemic conditions, chemotactic PMN trafficking and phagocytosis are significantly debased, resulting in exacerbated and prolonged inflammation [18, 19].

With regard to dental implant therapy, the T2DM-related biological alteration in wound healing may account for increased complication rates and even implant failures [6]. Accordingly, attempts to reduce the complexity and invasiveness of surgical procedures and to simplify the treatment regimen appear desirable.

Narrow-diameter implants (NDI) were developed for sites with diminished alveolar ridge dimensions and are a viable treatment option [20–22]. In T2DM patients, NDI may represent a less invasive treatment option by reducing the need for lateral ridge augmentation procedures and preventing an extended wound healing burden. Recently, in a 1-year clinical case and control pilot study, we were able to demonstrate the positive outcomes of NDI in T2DM patients [23].

However, the significant role of PMN in diabetic wound healing justifies the wish to better understand the role of PMN-related cytokines in this context. Here, we aimed to further substantiate the clinical performance of NDI in T2DM and non-diabetic patients by characterizing the process of wound healing in both study groups.

## Material and methods

### Study population

The study protocol was nested inside a 1-year prospective case–control trial [23]. The Witten/Herdecke University Ethics Committee approved the study protocol (108/2012) and the study is in accordance with the Declaration of Helsinki. All participants provided written informed consent and were compliant with the study protocol.

In brief, 32 patients with a mean age of 67 and one or more missing teeth posterior to the canine area of the maxilla or mandible and an associated diminished alveolar ridge dimension were enclosed (Table 1). Sixteen patients diagnosed with T2DM (HbA1C > 6.5%) were assigned to the test group and 16 normo-glycemic patients were assigned to the control group (HbA1C ≤ 6.0%). The individual HbA1c amount was determined by the patient’s physician before enrollment for the study. The absence of T2DM or prediabetes was also verified by consultation with the prospective participant’s physician. Exclusion criteria have been described previously [23]. A STROBE checklist for this study was provided for review of this manuscript.

**Table 1:** Patient demographics of all patients eligible for wound healing assessments. For the mean age of participants, the range is shown in brackets

	All groups	Test	Control	*p*
Number of patients	31	16	15	
Mean age (years)	67	70 (53–87)	65 (53–84)	0.08^✢^
Sex distribution (%)				
– Male (%)	14 (48.3%)	10 (61.5%)	5 (33.3%)	
– Female (%)	15 (51.7%)	6 (38.5%)	10 (66.6%)	0.29^✢^
Mean HbA1C (± SD)	-	7.34 (± 0.73)	5.3 (± 0.4)	0.0001^✢^
Jaw distribution				
– Maxilla	19	8 (5)	11 (6)	0.57^✥^
– Mandibula	29	15 (11)	14 (9)	
Number of implants	48	23	25	
Implant lengths				
– 8 mm	9	6 (2)	3 (3)	0.34^✥^
– 10 mm	23	10 (8)	13 (8)	
– 12 mm	16	7 (6)	9 (4)	

The numbers in brackets for implant length and jaw distribution signify the amount of study implants for this investigation

^✢^Student’s *t*-test

^✥^Fisher’s exact test

### Therapeutic intervention

All participants received hydrophilic-surface reduced-diameter tissue level implants (3.3 mm; RN Standard plus, SLActive, Institut Straumann AG, Basel, CH). By protocol, additional surgical steps aiming at the augmentation of bone volume were neither intended nor performed at the site of interest. Placement of all implants was carried out under local anesthesia (3.4 ml, Ultracain DS forte, Sanofi-Aventis, Frankfurt, Germany) and strictly complied with the manufacturer’s transmucosal healing protocol in both groups. All surgeries were carried out by one experienced periodontist (A.F.) in a standardized manner. After midcrestal incision, a buccal and lingual flap was reflected, strictly omitting vertical releasing incisions. Care was taken to maintain at least 2 mm of keratinized mucosa on both lingual and buccal flaps. The osteotomy was performed according to the manufacturer’s instructions. In cases of two adjacent implants, the posterior one served as the study implant.

The post-op regimen included instructions to abstain from mechanical plaque control in the treated area for 1 week and to use a 0.2% chlorhexidine mouth rinse twice a day (Chlorhexamed, GlaxoSmithKline Consumer Healthcare GmbH & Co. KG, Munich, Germany). The administration of systemic antibiotics was restricted to individual needs, and there was no prescribing policy by protocol; analgesic medication (Ibuprofen 600 mg/3 × daily) on demand was recommended. Sutures were removed after 7–10 days.

### Laser Doppler flowmetry

**Fig. 1:**
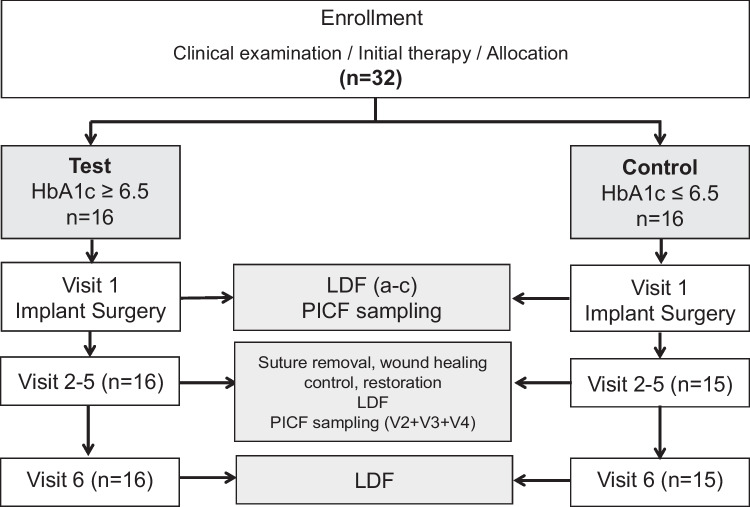
Flow chart of the study protocol. PICF, peri-implant crevicular fluid; LDF, laser Doppler flowmetry. Visit 2 = 3 days; visit 3 = 7–10 days; visit 4 = 4 weeks; visit 5 = 8 weeks; visit 6 = 3 months post op

A laser Doppler flowmeter (Periflux 5010, Perimed AB, Jarfalla, Sweden) equipped with a PF 416 probe (outside diameter 1.0 mm, fiber separation 0.25 mm; wavelength 780 nm) was used for the assessment of microcirculation. The scores were recorded in perfusion units (PU) and monitored by the Perisoft software (Perisoft 2.10, Perimed AB, Jarfalla, Sweden). To standardize the reproducibility of assessments, a customized acrylic stent fitting the retained teeth determined the positioning of the probe tip. The stent carried a perforation on the buccal aspect above and beneath the muco-gingival junction (MGJ). The perforation fitted the diameter of the LDF probe tip and coordinated both the perpendicular position of the tip at the mucosa surface and the distance of 0.5 mm above its surface. The stent was extended to the contralateral side and at the contralateral tooth the perforation was prepared according to the position at the implant. The microcirculation in peri-implant tissues was assessed above and beneath the MGJ before treatment (V1a), 2 min after local anesthesia (V1b), directly after completion of surgery (V1c), 3 days (V2), 7–10 days (V3), 4 weeks (V4), 8 weeks (V5), and 3 months (V6) post-surgery for 1 min each (Fig. 1). One calibrated investigator (A.B.) performed all measurements. Statistical analysis of perfusion units (PU) was carried out for the delta (Δ) between the baseline and following LDF measurements.

### PICF sampling

Sampling of peri-implant crevicular fluid (PICF, Fig. 1) was performed 3 days after surgery (V2), after suture removal (V3), and after 4 weeks (V4). In brief, a paper strip (PerioCol collection paper; Oraflow, Smithtown, NY, USA) was inserted to the sulcus on the buccal aspect of each study implant and kept in place for 10 s. Strips contaminated with blood were discarded. Fluid volume was determined immediately with a micro-moisture meter by means of a standard calibration curve (Periotron 8000, Oraflow, USA). Samples were stored in 200 μl sterile phosphate-buffered saline at − 80 °C until further processing. For elution, thawed samples were vortexed for 1 min and subsequently centrifuged at 3000 × g.

### Microsphere-based multiplex immunoassay

Reagent sets for Luminex immunoassay were customized by Merck Millipore and included four analytes (IL-1ß, GM-CSF, IL-17a, IL23). Immunoassays were performed according to the manufacturer’s Luminex magnetic screening protocol. The magnetic beads were dispensed into a 96-well microplate after blocking with washing buffer for 15 min. Fluid samples were normalized to a PICF volume equivalent of 0.1 ml (Table 2) and incubated with the mixed beads overnight at 4 °C. Beads were washed and incubated with biotinylated antibodies against the analytes for 1 h. Plates were washed again and streptavidin–phycoerythrin (PE) was added for 30 min with another subsequent washing step. Plates were read immediately on the MAGPIX (Luminex Corp., Austin, TX) instrument. Absolute protein concentrations were calculated in picograms per milliliter from a standard curve derived from a sevenfold (3.2–10,000 pg/ml) serial dilution of the manufacturer’s analyte standard.

**Table 2:** Mean PICF volumes in microliters at different time points stratified by group

		V2	V3	V4
Control	Mean	0.67	0.30	0.23
	SD	1.00	0.20	0.19
	Min	0.03	0.03	0.03
	Max	3.93	0.88	0.82
T2DM	Mean	0.64	0.35	0.25
	SD	0.53	0.60	0.21
	Min	0.09	0.03	0.09
	Max	1.77	2.56	0.93

### Statistical analysis

For all data obtained, mean and standard deviation were calculated. Raw data from luminex immunoassay were acquired from xPONENT (Luminex Corp., Austin, TX) software. All statistical analyses were performed with GraphPad Prism 8 (GraphPad, San Diego, CA). Censored values below the limit of detection (LOD) were replaced by a value between zero and the analyte-specific detection limit. In order to avoid overestimation of the statistical mean, half of the detectable LOD was employed using the formula (½)*(LOD − 0) [24]. Further statistical analysis included the Shapiro–Wilk, Kolmogorov–Smirnov, and D’Agostino-Pearson test to assess data distribution. Respectively, comparisons between independent samples were calculated by the Mann–Whitney *U* test and intragroup comparisons were analyzed by the Wilcoxon signed-rank test. LDF data were analyzed by two-way ANOVA and Sidak’s multiple comparisons post hoc analysis. The level of significance was set at *p* = 0.05.

## Results

The clinical outcome after the surgical intervention was reported previously [23, 25]. In brief, thirty-two patients with a mean age of 67 years were enrolled. Mean HbA1c value for the hyperglycemic test group was 7.34% (± 0.73). Thirty-one patients were eligible for wound healing assessments, as one patient from the control group was treated with systemic antibiotics for endocarditis prophylaxis (Table 1).

### Microcirculation

**Fig. 2:**
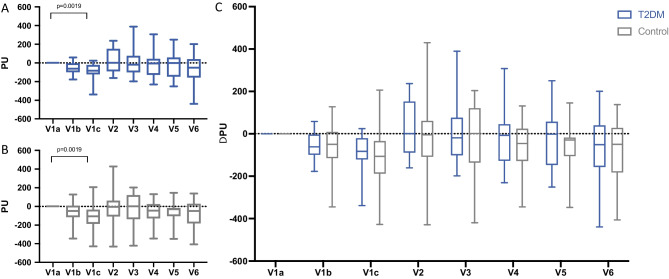
LDF measurements at different visits. Values represent the Δ of PU and the baseline measurement at V1a. Whiskers represent minimum and maximum. **A** Perfusion units at different visits in healthy control. **B** Perfusion units at different visits in T2DM patients. **C** Intergroup differences. V, visit

As shown by the Friedman test and consecutive Dunnet’s post hoc analysis, the LDF values in the control group experienced a significant reduction in perfusion rate immediately after completion of implant surgery (*p* = 0.0019) with a mean ∆PU (perfusion unit) of − 108.5 ± 142.5 (V1c, Fig. 2). This was followed by a consecutive increase in perfusion rate 3 days (V2) post-surgery (∆PU − 14.76 ± 185.0). However, this was statistically non-significant (*p* > 0.99). The consecutive LDF measurements revealed a non-significant diminution over the observation period (V3–V6, Fig. 2). In the T2DM group, ∆PU values exhibited a significant decrease at completion of the implant surgery (− 92.36 ± 87.40, *p* = 0.0019). At all further measurements, the development of site perfusion exhibited a pattern like the control group (Fig. 2C). Consequently, the intergroup comparison via Student’s *t*-test failed to show statistically significant differences at any visit and assessment, respectively (Fig. 2C).

### Cytokine quantities in peri-implant crevicular fluid

**Fig. 3:**
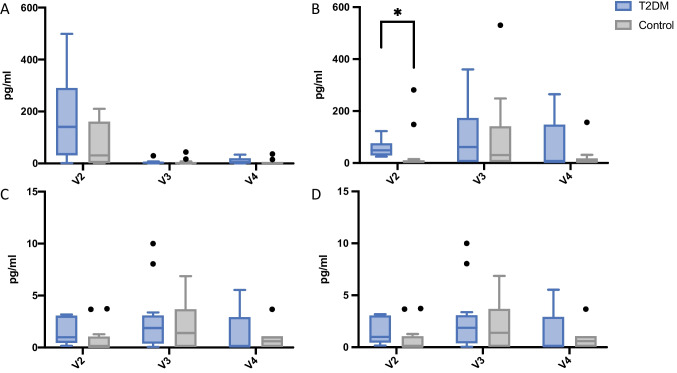
Analyte concentrations of IL-1ß (**A**), IL-23 (**B**), IL-17a (**C**), and GM-CSF (**D**) stratified by study groups. Whiskers represent minimum and maximum. Black dots signify outliers. *p <0.05

**Fig. 4:**
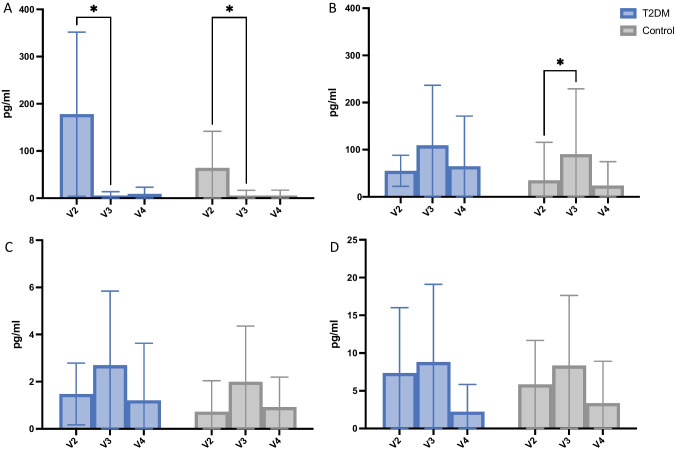
Analyte concentrations IL-1ß (**A**), IL-23 (**B**), IL-17a (**C**), and GM-CSF (**D**) stratified by time. Whiskers represent minimum and maximum. *p < 0.05

**IL-1ß —** At baseline, cytokine concentrations were significantly elevated compared with the subsequent visits in both groups (Fig. 3A), indicating a significant time-dependent reduction of IL-1ß in the PICF. Furthermore, the mean analyte concentration was substantially higher at implants of the T2DM group (178.05 ± 173.77) than in the control group (64.22 ± 77.42, Fig. 4A). However, these differences were not statistically significant (Table 3).

**Table 3:** Mean analyte concentrations in picograms per milliliter at different time points stratified by group and analyte. Significant values are indicated as bold

		T2DM		Control		Mean rank diff	Mann–Whitney *U*	*p*-value
		Mean	SD	Mean	SD			
**IL-23**	V1	55.16	33.05	34.98	80.54	7.86	16.00	**0.003**
	V2	108.96	127.67	90.59	138.58	1.76	76.50	0.82
	V3	64.76	106.48	23.83	50.63	1.11	23.00	0.82
**IL-17**	V1	1.48	1.31	0.73	1.32	5.71	18.00	0.09
	V2	2.70	3.14	1.99	2.37	1.99	88.00	0.79
	V3	1.21	2.42	0.92	1.27	-1.37	13.50	0.79
**IL-1ß**	V1	178.05	173.77	64.23	77.42	5.50	42.00	0.20
	V2	24.65	67.86	5.44	11.25	4.05	63.00	0.36
	V3	9.10	14.08	5.52	11.58	2.70	16.00	0.36
**GM-CSF**	V1	7.36	8.65	5.85	5.83	0.39	54.00	0.99
	V2	8.81	10.30	8.34	9.28	0.07	101.50	0.99
	V3	2.23	3.62	3.37	5.55	-0.67	22.00	0.99

**IL23 —** Stratified by study group, the baseline cytokine concentrations exhibited significantly higher amounts (*p* = 0.003) in the T2DM group (55.16 ± 33.05) than in normo-glycemic group implants (34.98 ± 80.54) (Fig. 4B). In relation to the process of wound healing, both groups exhibited a similar (*p* = 0.82) increase 10 days after surgery (V3, Table 3). These changes were statistically significant in the control group.

**IL-17 —** In total, the cytokine concentrations of IL-17 were lower compared to the other analytes, ranging from 0.73 to 6.44 pg/ml. No significant differences were detectable, neither stratified by time nor by study group (Figs. 3 and 4, Table 3).

**GM-CSF —** No significant changes in cytokine concentration were found during the entire observation period. Moreover, test and control group implants displayed no different levels of GM-CSF.

## Discussion

The present prospective clinical study was conducted to monitor the wound healing after placing narrow-diameter implants in T2DM and normo-glycemic patients by assessment of microcirculation and PMN-related cytokine expression. No significant differences between T2DM and healthy patients were observed, except for pro-inflammatory cytokines IL-23 and IL-1ß during initial healing a few days after implant surgery.

In our study, the perfusion of the peri-implant tissues was evaluated by laser Doppler flowmetry (LDF), a technique which allows for the detection of blood flow disturbances after surgical injury [26–28]. Both groups displayed significant reduction of microcirculation activity directly after surgery and 1 day post-op, when compared to baseline. However, microcirculation activity appeared to recover rapidly, returning to levels assessed before injury. Moreover, there was no significant difference regarding reduction of blood flow found between T2DM and healthy patients. The rationale behind this observation may be the similarly minimal-invasive surgical approach in both groups, since LDF measurements of post-surgical perfusion impairment were shown to correlate well with the extent of tissue trauma [28, 29]. The transmucosal healing pattern may have contributed to this positive outcome, as there was no need in flap closure above the implant shoulder, which regularly requires a coronal advancement of the flap. The perfusion rates altered at similar altitude during the observation period closely reflecting the healing progress in both groups. Statistically significant impairment of perfusion was only found before and after application of local anesthesia containing the vasoconstrictor in both groups. Moreover, the perfusion rate showed statistically significant improvement at day 3 after the surgery. Regardless of systemic background, the recorded microcirculation was apparently related to uneventful healing progress. Furthermore, the plotted perfusion units per time were in accordance with those observed earlier in a beagle model that aimed at monitoring the changes of microcirculation in the context of bone augmentation surgery [26].

These results, however, contradicted our expectations. As suggested by recent studies on the microvascular skin perfusion in diabetic patients, we anticipated a significantly lower perfusion rate in the wounds of diabetic patients [30, 31]. The Hba1c values in the T2DM patient cohort with 7.34% might have been estimated to cause a bigger discrepancy. Nevertheless, the results highlight our hypothesis that T2DM patients may benefit from the NDI design because of the reduced wound healing burden associated with their use [32].

In this study, the concentrations of interleukin-1ß, interleukin-23, interleukin17A, and GM-CSF were measured in PICF of NDI recently placed in the posterior jaw. The sampling of the PICF for analyzing the molecular content is considered a viable method for studying the wound healing biology around teeth and implants in humans [33–35]. In particular, the bead-based immunoassay applied in this study allows for a multiplex analysis of up to 96 analytes per sample.

Interleukin-1ß is a pro-inflammatory cytokine which upregulates a plethora of inflammatory effector molecules such as chemokines and prostaglandins. Due to its abundance in inflamed and injured tissues of all kind, it is considered a ubiquitous biomarker for acute tissue inflammation [12, 36]. This study revealed upregulated concentrations of IL-1ß immediately after surgery presenting with significantly reduced levels up to the end of the observation period. Interestingly, the T2DM patients presented with non-significantly altered concentrations to the control group at any of the visits, indicating both an innate initialization and resolution of the inflammatory process, respectively. The pattern of IL-1ß secretion to PICF is in line with a similar study conducted by Guarnieri et al. [37], who reported similar results for IL-1ß concentrations in PICF of healthy patients.

According to a recent experimental study in diabetic rats, IL-23 levels are significantly increased under diabetic conditions [38]. IL-23 is considered the main inducer of CD4 + T-lymphocyte differentiation towards IL-17 producing Th17 cells [17]. In context of wound healing, large amounts of Th17 helper cells are associated with a delay in wound closure. The inhibition of the backbone IL-23/IL-17 axis may even promote wound healing under diabetic conditions [39, 40]. Our results demonstrated significantly higher levels of IL-23 at implants of T2DM patients, when compared to the control group. Over the course of the observation period (V2-V4), however, the IL-23 and IL-17 concentrations equalized to insignificant differences. These results are corroborated further by the works of Santos et al. and Vieira Ribeiro et al., who reported no significant increase in IL-23 expression among diabetic and healthy patients suffering from periodontitis [41, 42].

The last investigated analyte, GM-CSF, did not show any timing- or group-related differences. GM-CSF is a multipotent growth factor responsible for granulopoiesis and keratinocyte-related proliferation and re-epithelialization, and a variety of studies have verified its necessity for complication-free wound healing [43–45]. Therefore, unchanged GM-CSF levels in T2DM patients may have been associated with the uneventful healing period observed in this trial.

This analysis was nested in a larger pilot study [23]. Thus, the presented results have some obvious limitations. As indicated by the large variability among individuals, selected datasets may not be adequately powered. Nevertheless, within this frame of limitation, we were able to elucidate that the wound healing profile of NDI in T2DM patients resembles that of healthy individuals in terms of blood perfusion. Moreover, the measured effector molecules have a pivotal role in regulating the PMN-mediated early wound healing, which did not appear significantly affected by the T2DM condition. However, while diabetic patients showed a more pro-inflammatory initial cytokine profile, wound healing and PMN response appeared similar in diabetic and non-diabetic patients on a longer term. Altogether, these results corroborate our previously reported findings of similar clinical success placing reduced-diameter implants in these patients [20]. In conclusion, hydrophilic-surface titanium-zirconium implants with reduced diameter exhibit no molecular alterations regarding wound healing in T2DM patients and may thus be a viable option for this group of patients.
